# Lockdown Experiences and Views on Future Research Participation of Autistic Adults in the UK During the First 6 Months of the COVID-19 Pandemic

**DOI:** 10.1089/aut.2022.0027

**Published:** 2023-08-30

**Authors:** Alba X. Realpe, Nicola Mills, Lucy Beasant, Sarah Douglas, Lorcan Kenny, Dheeraj Rai

**Affiliations:** ^1^ Department of Population Health Sciences, Bristol Medical School, University of Bristol, Bristol, United Kingdom.; ^2^ Autism Study Advisor, Bristol, United Kingdom.; ^3^ Autistica UK Charity, London, United Kingdom.; ^4^ Learning Disability and Autism Programme, NHS England & NHS Improvement, London, United Kingdom.; ^5^ Avon and Wiltshire Partnership NHS Mental Health Trust, Bath, United Kingdom.; ^6^ NIHR Bristol Biomedical Research Centre, University Hospitals Bristol and Weston NHS Foundation Trust and University of Bristol, Bristol, United Kingdom.

**Keywords:** autism in adults, COVID-19 pandemic, first lockdown experiences, qualitative study, trial methods

## Abstract

**Background:**

The COVID-19 pandemic resulted in large-scale public health restrictions and lockdowns across many countries. There is an increasing literature on the varied impact of such lockdowns in autistic adults. However, there is very little research on how the pandemic and related public health measures may impact the willingness of autistic people in engaging and taking part in research. The aim of this qualitative study was to explore autistic adults' experiences of the COVID-19 lockdown and how the pandemic may affect future research participation.

**Methods:**

We conducted in-depth interviews with 31 autistic adults between March and July 2020. Transcripts were analyzed thematically within a critical realism framework.

**Results:**

Participants identified positive aspects of lockdown such as enjoying the lack of social pressures and using their well-developed skills for dealing with uncertainty. Autistic people also shared challenges of adjusting to lockdown, for example, rapid change in daily routines. While hopeful about the freedom gained from easing restrictions, participants were concerned about the inconsistent communication and application of rules during the transition out of lockdown. This may have exacerbated already rising mental health issues among autistic people. The participants viewed research participation and engagement with increased relevance during the pandemic and welcomed efforts to conduct research using online methods of communication.

**Conclusion:**

The COVID-19 lockdown had a varied effect in the lives and routines of autistic people. However, health care providers and researchers need to be mindful of rising mental health issues in the aftermath of the pandemic, especially for people who were already vulnerable. The response to the pandemic may have offered opportunities for innovation in research processes enabling more autistic people to engage with research and making studies more inclusive.

## Community brief

**Why is this an important issue?:**

We did not know how the pandemic and the strict restrictions that followed would affect autistic people's well-being and mental health.

Also, there was a worry that the pandemic would affect the number of volunteers taking part in research that matters the most to autistic people. Thus, it was important to understand any implications for the way we conduct research with the autistic community after the pandemic.

**What was the purpose of this study?:**

We explored the experiences of autistic people living through the first 6 months of the COVID-19 lockdown in the United Kingdom. We were particularly interested in autistic people's views on how the pandemic may affect them taking part in research.

**What did the researchers do?:**

We co-produced this interview study to answer our research questions. We carried out in-depth interviews with 31 autistic people. We looked for patterns or themes in what the participants said.

**What were the results of the study?:**

Autistic people we interviewed reported being able to enjoy a quieter pace of life. They felt less anxious early in the lockdown. But they also faced great challenges adjusting to changes in their daily routines. Inconsistent public health communication caused worry during the transition out of lockdown. Unnecessary stress might have led to worsening of mental health issues in some people. Our participants held positive views on taking part in and engage with research, despite the pandemic. We identified opportunities that could make research more inclusive for autistic people, for example, online methods for taking consent and taking part in research remotely.

**What do these findings add to what was already known?:**

Our study adds to the evidence of the varied responses of autistic people to the pandemic and the public health measures that it led to. One important strength of our work is our focus on the impact of the pandemic on research and implications of future research. We learnt that autistic people welcome and value the use of online technology to reach study participants. Wider use of remote technology can make research more inclusive and participatory.

**What are potential weaknesses in the study?:**

Many of our participants were already had experience participating in research. Also most had relatively high education levels. We did not include autistic people with intellectual disabilities. We did not collect information on ethnicity. Our sample is likely to have little ethnic diversity.

**How will these findings help autistic adults now or in the future?:**

We describe the experiences of autistic people in the face of unprecedented circumstances. We found the need for clear public health communication to avoid unnecessary stress. The pandemic has provided the opportunity for a wider use of remote methods of research, even in areas where this was not done in the past (e.g., clinical trials). Our study found that such approaches would make research more inclusive.

## Introduction

Following a rapid rise of COVID-19 cases, the UK government imposed a set of public health restrictions, including limits on social mixing, business closures, and guidance to maintain social distance and wear face coverings (hereafter, COVID-19 lockdown) in March 2020.^1^ Global research efforts gathered pace to understand the behavioral and social impact of lockdown strategies on different communities using large surveys and systematic reviews in various countries.^2–4^ Emerging evidence indicated that strict restrictions may have had a negative impact on people's well-being and mental health,^2,4^ especially in individuals living with pre-existing mental health conditions.^5–7^

Public health researchers considered the autistic community at risk of experiencing hardship during the pandemic^8^ and particularly vulnerable, due to high rates of pre-existing physical and mental health problems.^9–12^ Various online surveys, mixed-method and qualitative studies, published since, have documented the experiences of autistic adults in different communities in the United States,^13,14^ Australia,^15,16^ and various European countries,^17–24^ including the United Kingdom.^25–29^

These studies described negative effects of the pandemic and lockdowns that included increased feelings of anxiety, stress, and sadness; varied levels of disruption to autistic adults' daily routines; and difficulties accessing professional support services. However, studies also reported that some autistic adults benefited from reduced social and sensory stimulation,^16,17,20,28^ recording better than expected outcomes based on lower expectations for social interaction leading to less perceived stigma and exclusion.^19,23^

Autistic people and their families deployed a variety of coping mechanisms to reduce anxiety and uncertainty^20,25^ and developed increased community spirit during the COVID-19 lockdown.^15^ Further, the rapid replacement of face-to-face services for online/telehealth represented a unique opportunity to improve services' reach and overdue adaptations to the needs of the autistic community.^18,29^ However, there has been less research in relation to the implications of the pandemic in relation to the conduct of future research with the autistic adult population.

### Research participation and the autistic community

Autistic people often have extensive unmet health care needs,^30^ yet effective care will require a strong scientific evidence base. The autistic community has frequently raised concerns about researchers not focusing on the community's needs and priorities.^31,32^ Further, community-based participatory research has shown there is a tendency of investigators to implement research processes (e.g., use of validated instruments, attending study appointments) without considering autistic people's neurodiversity and their unique cognitive, behavior, and sensory needs.^33^

Despite high levels of motivation to take part in research, autistic people may feel extremely anxious, confused, or angry when confronted with inappropriate research processes and instruments, discouraging participation and bearing on the validity, reliability, and real-world meaning of autism research.^34,35^ In response to these challenges, research partnerships between academics and autistic community members, such as the Academic Autism Spectrum Partnership in Research and Education in the United States,^31^ have proposed guidelines^32,36,37^ to engage autistic people in the whole research cycle.

Researchers within these partnerships have also identified autism-specific adaptations to research participation that include, for example, embedding flexible modes of participation within clear processes, respecting the need for personalized adjustments to daily routines, and creating trust based on understanding and willingness to share power.^33,34^

However, we know less about engaging autistic adults and community preferences in relation to randomized controlled trials (RCTs). To address this gap, we were conducting detailed qualitative interviews about research participation (in RCTs) with autistic adults when the COVID-19 pandemic broke out, and we took the opportunity to investigate how the pandemic might affect autism research.

The aims of the present study were therefore: (1) to understand the experiences of autistic people of the first COVID-19 UK lockdown and how they impacted their daily lives; (2) to explore the views of autistic people on how the pandemic may affect future research participation; and (3) to understand which adaptations may be helpful to facilitate research participation during and after the pandemic.

## Methods

The study design was qualitative with in-depth one-to-one semi-structured interviews with autistic adults. Critical realism informed our methodological approach, which proposes that all scientific knowledge is historical and produced within a specific sociocultural context. Therefore, the aim of any investigation is to create a plausible description or explanatory account of the object of study.^38^ We also subscribe to the view that the exploration of people's speech and behavior is a valid method to find out how they exercise personal agency in a socially coordinated and interdependent manner,^39^ thus justifying our interview approach.

Our study team comprised an autistic adult (S.D.), the head of research at the UK charity Autistica (L.K.), experienced qualitative researchers (A.X.R., L.B., N.M.) in RCT methodology, and an experienced autism clinician and researcher (D.R.). We co-produced the study design and all study materials. We obtained ethical approvals for the original Autistic People and Randomized Controlled Trials (APRiCoT) study and an amendment to include questions about autistic people's lockdown experiences from the University of Bristol Faculty of Health Sciences Ethics committee (ref. 93283/August 18, 2019 to May 20, 2020).

### Participants

Participants were individuals who were 18 years or older, had a diagnosis of autism, understood the participant information sheet, and consented to participate in a 1:1 interview with a researcher in English.

We interviewed 31 autistic adults between May and July 2020. Table 1 shows their demographic characteristics. Fourteen people identified as females, 15 as males and 2 as non-binary. Most interviewees were working-age adults with higher levels of education attainment, yet almost half of the participants were either under- or unemployed. All participants confirmed they had a diagnosis of autism. Interviews lasted on average 54 minutes (range 23–105 minutes) and conducted via an online platform (*n* = 13, e.g., Skype, Bluejeans, Zoom), telephone (*n* = 12), email (*n* = 5), and text messaging (*n* = 1), depending on participant preference.

**Table 1.** Demographic Characteristics of the COVID-19 Lockdown Interview Sample

Characteristic	Value (%)
Age (range)	45 (25–67)
Gender	
Female	14 (45)
Male	15 (48)
Non-binary	2 (6)
Highest education qualification	
Pre-degree	9 (29)
First degree	11 (35)
Postgraduate	8 (25)
Current occupation	
Full time	11 (35)
Part time	5 (16)
Retired	5 (16)
Student	4 (12)
Unemployed/long term disability	6 (19)

*Note*: Age = Mean age. Pre-degree qualifications includes certificates of secondary education and vocational qualifications. First degree includes Bachelor of Science or Art. Postgraduate includes Master and PhD. Other = volunteering, long term absence on medical grounds.

### Procedures

We started a qualitative study of the views of autistic people in relation to RCTs and their processes (the APRiCoT study, paper in preparation) as preliminary work toward the set-up of a randomized trial of anxiety treatment (bristol.ac.uk/strata). We were open for recruitment when the COVID-19 pandemic struck. Autistic adults who had subscribed to hear about potential research opportunities from the UK charity Autistica, which links researchers to individuals interested in participating in research, received an invitation via email.

We also posted a call for participants through social media. Interested potential participants completed an expression of interest confirming their diagnosis of autism, understanding of the participant information sheet, and providing basic demographic information, and preferred contact details for an interview in English. We invited a subset of those who expressed an interest for an interview. We aimed at achieving a participant group balanced in terms of age, gender, level of education, and employment status to ensure a maximum variation.

An experienced qualitative researcher (A.X.R.) approached potential participants to obtain informed consent and organized interviews remotely according to participant preferences. Four members of the study team (A.X.R., L.B., N.M., D.R.) conducted the interviews. We used a semi-structured topic guide that included questions about participants' COVID-19 lockdown experiences (e.g., *How has [the coronavirus crisis] affected you and your routines/social interactions? How does being autistic affect the way you have coped with the current pandemic?*) and views on research participation during and after the pandemic (e.g., *are you the kind of person who would usually volunteer to take part in research? Has this view changed in any way due to the coronavirus pandemic?*).

For the purpose of this article, we are reporting responses to Part A, Part B and questions relevant to the COVID-19 pandemic in Part D of the topic guide. See Supplement 1 in Supplementary Data for a complete list of questions. We carried out interviews between March and July 2020 and prepared transcripts at verbatim for analysis.

### Qualitative data analysis

Our analytic approach followed principles of reflexive thematic analysis.^40,41^ Interviewers approached the interviews with curiosity and somehow naive expectations because, with exception of D.R., they had either limited or no experience talking to autistic people. However, we all shared our experience of an unprecedented public health crisis that the COVID-19 pandemic represented, which created deep and meaningful exploration of lived experiences for interviewees and interviewers alike. After reading interview transcripts repeatedly, two authors (A.X.R., L.B.) developed initial codes from an inductive perspective assisted by specialized software NVIVO (released on 2018).^42^

Researchers met regularly to build themes from the initial coding, refining them, and discussing disagreements. The wider team discussed ongoing analysis at regular meetings, in particular to engage on reflexivity.^41^ Conversations between members reflected on how our different professional (research methods, clinical, advocacy) and personal (e.g., neurotypical vs. neurodivergent) backgrounds shaped differences and similarities of opinions about positive and negative experiences of the pandemic, and views on research participation during and after the public health crisis.

Sampling, data collection, and analysis occurred cyclically until we considered we had enough information power^43,44^ to support our proposed categories and themes structure, which in our view reflected a nuanced and rich description that answered our research questions.

## Results

**FIG. 1.**
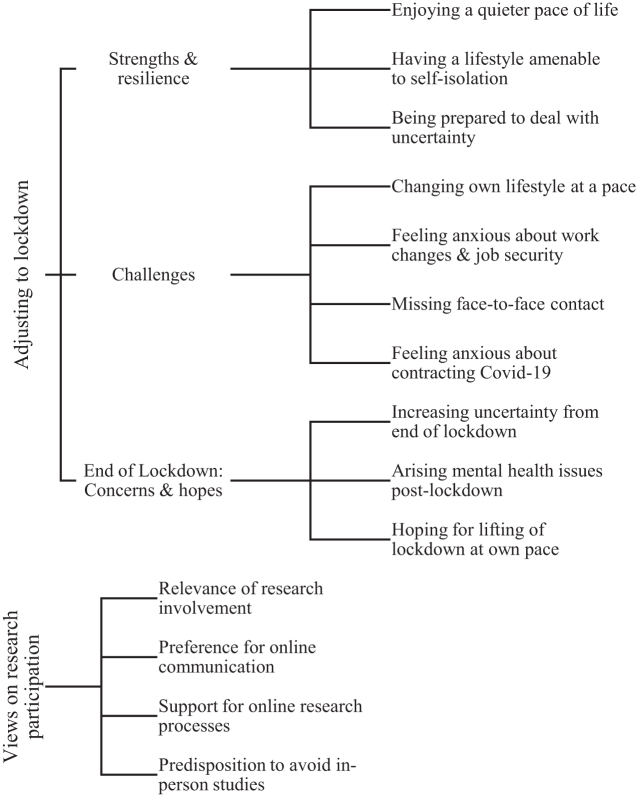
Themes by categories from study interviews (*N* = 31).

We report the 31 study participants' experiences adjusting to the conditions of lockdown in themes under three categories that detailed (1) their perceived strengths and resilience, (2) challenges they faced, and (3) their concerns and hopes for life after the first lockdown. In relation to research participation, we organized participants' views under four themes that reflect perceived opportunities and barriers for research engagement of autistic adults. Figure 1 shows the categories and themes structure derived from our analysis. We included quotes to exemplify the findings described later. Further quotes (Supplement 2) and a list of the raw codes (Supplement 3) are available in the Supplementary Data.

### Adjusting to lockdown: strengths and resilience

Most autistic people in our interviews viewed themselves as particularly well-equipped to deal with the lockdown. This was, in part, due to their lifestyle preferences of rules and avoiding social interaction, and in part because participants already dealt with higher levels of uncertainty and anxiety in their everyday lives. We described the factors cited later as contributing to autistic people's positive perceptions of themselves during lockdown.

#### Enjoying a quieter pace of life

The quieter pace of life and reduced social expectations during lockdown suited several participants. Pressure to socialize reduced during the lockdown, often a source of anxiety for autistic people. People reported having more time to work at a measured pace and engaged with physical exercise and leisure activities (e.g., hobbies). Public spaces were emptier with rigid rules (such as one-way systems and social distancing), so activities such as shopping were less stressful:

“All the stressful events were removed and replaced with things I was choosing to do. I settled into it very quickly and enjoyed every minute of it. I was never bored and kept busy with lots of things.” [P45]

#### Having a lifestyle amenable to self-isolation

Participants reflected on already having a lifestyle amenable to self-isolation:

“I'm very well-suited to COVID life. I spend a lot of [time] at the house by myself anyway. I've got quite insular hobbies, so I like writing, I like painting.” [P37]

To avoid anxiety from unexpected changes, many people had deliberately designed their lives to have less social interaction. For example, they preferred a small group of friends and already worked or study on their own and from home. Many were already carrying out everyday activities, such as socializing or shopping, via online platforms. An advantage of communication using digital platforms during lockdown was that people were more available.

#### Being prepared to deal with uncertainty

Most participants had developed some resilience to deal with uncertainty pre-pandemic. They had experience of managing anxiety related to uncertainty and told us how this helped them to adapt to the pandemic crisis; for example, by having a pragmatic view of events and situations, focusing on what they can control and using problem-solving skills. Some reported that their natural tendency not to react emotionally was an advantage during this crisis:

“I'm quite matter of fact. So, I don't particularly often have very strong emotions. So, I tend to look at it and look at what the science is saying and what's happening, and take quite a pragmatic view, from that point of view. And I think that's probably due to the autism.” [P29]

One participant reported a caveat to the positive experiences and attitudes to lockdown when reflected on how positive attitudes depended on having an overall good mental health pre-pandemic:

“As long as the particular autistic person has good mental health, I think [lockdown] probably isn't that much different [to life before] apart from it's a bit quieter when you go outside. So, yeah, I mean luckily my mental health is quite good so that is not an additional thing to worry about. And I think from what you see with neurotypical people, they are missing the things like the pubs and football matches and the more present social things that they like to do” [P44].

### Adjusting to lockdown: challenges

All autistic people in our sample described the challenges affecting their physical, psychological, and social well-being during the lockdown measures. We describe these challenges and present quotes to exemplify the findings given next.

#### Changing own lifestyle at a pace

For many participants, changes brought about by the lockdown interfered with daily routines they employed to feel safe and secure; these increased feelings of anxiety and, in some instances, caused a low mood. Some people struggled to create new routines to replace old ones, especially at the beginning of the lockdown measures. A few people had to change plans (e.g., postpone a wedding or move in with relatives to take on caring duties):

“The first 3 weeks were incredibly difficult. All those micro routines which completely went out of the window, Including the times that I get up. The time that I start and finish work. It completely went out of the window, and it just flawed me. There was no warning (…) So, [lockdown] has had a huge impact because, all those little routines that you have, they create a feeling of safety and wellbeing.” [P27]

Further, some felt preoccupied by the planned lifting of lockdown restrictions because they anticipated a new wave of rapid changes to their newly adjusted routines. Adjusting their lifestyle to different regulations in succession did not suit autistic adults and their families.

#### Feeling anxious about work changes and job security

Although most participants had experience working from home or were retired, most interviewees commented on raising levels of work-related stress because of changes in working patterns and conditions, and some people were not used to working from home and missed their commute routine. Half of the participants were either under- or unemployed, which is representative of what is generally found in the autistic population.^45^ Three people felt their current jobs exposed them to a higher risk of contracting the virus. Four people had lost their job as a result of the pandemic, placing them in financial hardship:

“I'm a bit of a loss as to where to go at this stage in terms of finding work, what to do or what would be available.” [P49]

#### Missing face-to-face contact

Autistic adults living on their own shared their feelings of loneliness and longing for meeting people face-to-face and for physical contact (e.g., hugs):

“So, I've kind of seen a few friends. But I cry when I first see them because I can't hug them. Because I'm a very tactile person. And I really love hugs.” [P41]

They not only missed their close friends but also casual regular acquaintances. One female participant commented on feeling surprised by her own proactivity chatting to neighbors during the ‘clap for carers' events and obtaining a phone number from someone at a coffee shop; behaviors she would not engage in voluntarily before lockdown measures. Social interaction via online platforms offered ways to maintain social contact with significant others; however, four people commented on barriers such as difficulties recognizing social clues, for example, to be able to take turns in a conversation or maintaining eye contact.

In contrast, people sharing accommodation with others reported feeling overwhelmed by the constant social interaction and found it difficult to take time out to rest although to a small degree.

#### Feeling anxious about contracting COVID-19

A few participants expressed fears about contracting COVID-19 that made them feel vulnerable and anxious, some of them were concerned enough to avoid going out at all at the beginning of the lockdown measures, and one had contracted and recovered from the virus. Another participant felt that their personal risk was low on account of good health, youth, and fitness.

About half of the participants reported concerns about family and friends during the pandemic. They worried about vulnerable people (e.g., older relatives) contracting the virus but also the indirect consequences of the lockdown measures (e.g., access to urgent health care, job losses). Autistic people expressed understanding of the stress and challenges experienced by neurotypical people and had concerns about not being able to provide support to significant others, for example, by sharing emotional experiences with them:

“My wife finds that she's feeling quite emotional, I can be a bit matter of fact. It's helpful for me. Not necessarily helpful for people I'm living with. I'm not sure I can always give the support (laughs) that she wants.” [P29]

### End of lockdown: concerns and hopes

We recorded concerns about the easing of lockdown restrictions and hopes for the new “normal” post-lockdown world.

#### Increasing uncertainty from lifting of lockdown

Some participants shared concerns and were anxious about the uncertainty that lifting restrictions would produce in their everyday lives. First, participants worried about people not following safety protocols (e.g., social distancing), which contrasted with their own strict adherence to guidelines:

“Even when I try and go out on a walk, I get myself so stressed because people are so blasé, they don't care.” [P26]

Second, autistic people found government guidance ambiguous about what was and was not allowed (e.g., visiting people) and felt very frustrated by the inconsistency, lack of clarity, or precision. Third, participants anticipated the return of previous anxiety-provoking routines such as inflexible working patterns or unwanted social interaction. Finally, they worried about uncomfortable sensory issues from face coverings and hand gel usage.

#### Arising mental health issues post-lockdown

Some autistic people recognized the potential for mental health issues arising post-lockdown. They wondered about the detrimental effect of increasing uncertainty from the lifting of restrictions. Further, some participants had postponed access to health services because of the pandemic and were unsure about services resuming:

“I think initially I went into kind of crisis mode and for about a month and a half I functioned really well. And I think it hit me in a sort of delayed - So, the past few weeks have been a lot more difficult than even initially.” [P48]

#### Hoping for lifting of lockdown at own pace

Participants expressed their hopes for understanding of the challenges that rapid changes in routine represent for autistic people. They wished for organizations and systems to embed flexibility and time for readjusting to a new way of working and interacting. These hopes extended to key public services such as social and health care, for example, by continuing online delivery as well as provision for safe return to face-to-face contact when required:

“I have a lot of things like medical and physical stuff for me and my son (…) And as things go more back to normal, I'm due to have a whole load of stuff now that's had to wait. Suddenly get - actually accelerated a bit and it's that part that I'm worried about - is like now go from this really quite quiet and for me quite easy life to full throttle - back to lots of things to feel anxious and stressed about all the time.” [P37]

### Views on research studies during and after the current pandemic

Most participants expressed positive views on research engagement and participation going forward. They commented on specific opportunities and challenges brought about by the pandemic.

#### Relevance of research involvement and participation

Most autistic people considered research involvement and participation more important now than ever “a result of this pandemic, people will have opened their minds to more research (…) that everybody has to look after everyone else” [P37]. They believed contributing to research was a citizen duty and society should encourage participation. They felt strongly about autism research and that this was a way to combating marginalization and improving the mental health and lives of autistic people:

“I think that in general the Coronavirus pandemic has shown that it's important for as many people as possible to participate in studies, if they possibly can, because the bigger your dataset the more accurate your information will be.” [P36]

They reported that the COVID-19 pandemic did not discourage them from participating in future research; however, they were aware of necessary adaptations. One participant warned about the need for coordinated efforts to prevent overlapping research, whereas another participant noticed a tendency to overwhelm potential participants with requests, assuming people were always at home. Many people we interviewed had a good knowledge and previous experience of research participation.

#### Preference for online communication

Online communications opened opportunities to participate in research according to most participants. The current pandemic has forced research teams designing or conducting complex studies (e.g., RCTs) to seek other ways to reach participants. One person saw this as an opportunity to innovate rather than persisting with a one-size-fits-all in-person appointments:

“Choice is key here really. Phone, skype, emailing etc. some studies have even been done via Facebook messenger. But each autistic person will want different, so I think it's best not to prescribe a method. And asking autistic people if there's other better ways than have been offered.” [P30]

Half of the participants reported being unable or unwilling to engage in research before the pandemic due to increased anxiety from meeting new people in an unfamiliar place, particularly clinical settings, but they would be more receptive now given the opportunity of online communication.

Participants had a variety of preferences to communicate online in a research context, from written communication (texts and emails) to videoconferencing and telephone. A minority still preferred face-to-face interaction and reported difficulties with interruption and fatigue when using videoconference platforms:

“Seeing somebody face-to-face is great and I've no problem with that. But for some reason video conferencing is just—urgh. It's really hard work (…) there's a lot of talk about Zoom fatigue.” [P27]

#### Support for online research processes

Autistic people supported changes to obtain informed consent via online contact. People we interviewed thought that verbal consent or consent documented via electronic documents were as valid as written consent, providing researchers observed data safety processes:

“But how accessible [the research] is, it is a major one [factor]. If it's online, I'm more likely to do it. I would only do one in person if it were at one of my two local unis within walking distance. And I usually have childcare issues for that, so can only go at certain times.” [P40]

One person was reluctant to give online information due to privacy concerns, but others thought it was feasible and even preferable for convenience and accessibility. However, they felt it was important to see and/or hear a person at the beginning, and not solely relied on electronic documents.

#### Predisposition to avoid in-person studies

Most autistic people said face-to-face studies carried more risks during the pandemic. Social distancing added to the stresses of meeting new people in unfamiliar places; therefore, studies that required in-person attendance would need to justify reasons for these extra risks:

“I'm aware from my knowledge of the history of pandemics that hospitals are not particularly good places to be. One of the pieces of advice was always ‘don't go to hospital if you don't have to,’ so I would probably be more wary now of about face-to-face [research activity]” [P32]

They were less inclined to take part unless they were drug or medical intervention studies, which required specific procedures (e.g., blood tests, scans, etc.). To see participants face-to-face, researchers would need to be well resourced; that is, better communication, increased staff capacity to explain COVID procedures, possibly more flexible reimbursement protocols.

## Discussion

In this study, autistic adults identified positive aspects of the first COVID-19 UK lockdown such as enjoying the lack of social pressures and using their well-developed skills for dealing with uncertainty. They also shared challenges of adjusting to lockdown, for example, due to the rapid change of daily routines. Although participants were hopeful about the freedom gained from easing restrictions, they were concerned about the inconsistent communication and application of rules during the transition out of lockdown, which may exacerbate mental health problems in autistic people.

The participants viewed research participation and engagement with increased relevance following the pandemic and welcomed efforts to conduct research using online methods of communication.

To our knowledge, our results are comparable to findings of four other studies that reported qualitative data from open response survey questionnaires^15,20,25,27^ and in-depth interviews with members of the autistic community in Australia,^16^ the United Kingdom,^29^ and Sweden.^23^ Similar to our study, others have reported a wide range of positive and negative effects of national lockdowns in everyday experiences of autistic groups in the United Kingdom, Belgium, the Netherlands, Sweden, Spain, Italy, the United States, and Australia during the first months of the COVID-19 lockdown.

These international studies as well as a recent report by the National Autistic Society^46^ confirmed the concerns from participants in our study about the increasing levels of anxiety, worsening mental health, and urgent need for support. The economical and societal consequences of the pandemic are unfolding and risk widening pre-existing health inequalities for people with disabilities.

Our study will add to the record of what was learnt during the COVID-19 lockdowns, for example, the need for clear and unambiguous government guidance, more flexible working practices that consider individual needs and preferences (such as remote working), and a system that values the needs of a neurodiverse population.^47^

### Research participation during and after the pandemic

Although research to date has focused on health care access and support needs, our study adds to the growing evidence by specifically investigating the implications the pandemic may have on future research with the autistic community or adaptations required to support research participation. In relation to research participation and engagement, we learnt that autistic people welcome and value the current use of online technology to reach study participants.

This finding is consistent with principles to promote research engagement identified by Haas et al^33^ and Nicolaidis et al.^36^ Online access provide flexible opportunities to participate from a safe and calm environment (i.e., home), which fit participants' daily routine and could be easily rescheduled for their convenience. Although it may be tempting to assume that all autism research should be online, we also found that online methods may not satisfy some autistic people who may prefer face-to-face contact with health care professionals, especially in relation to research involving RCTs of medication and other complex interventions, depending on the risk profile of a given research study.

It is in this aspect where trust with researchers may play a role, yet we need more research on this factor. Nevertheless, our research supports the view that in relation to accessibility, researchers must avoid a ‘one size fits-all’ approach^33^ and instead we should embrace strategies that welcome diversity and sensibility, reflecting the unique profile of autistic people and with the aim of achieving participatory autism research.^36^

That means adapting the research environment, methodology, and dissemination routes to permit the widest and most accessible engagement.^37,48^ The response to the pandemic provided an opportunity to accelerate innovation toward this aim by showing researchers ways to make research more accessible that takes into account the participant's experience, a finding that we anticipate could have implications not only to autistic people but also to all research participants. Our findings helped inform the design of an RCT with autistic adults to determine whether Sertraline is an effective treatment for anxiety (STRATA RCT bristol.ac.uk/strata).

### Limitations

Four features of this work limited the conclusions we can draw about the impact of the lockdown on autistic adults. First, participants' views and experiences represented an initial response to the first COVID-19 lockdown and therefore provided cross sectional data. However, we noted how the group response changed as restrictions eased (e.g., increased anxiety about inconsistent communication) even during the brief period of data collection, indicative of a dynamic situation; research from later in the pandemic started to provide a more comprehensive picture of the effects of consecutive lockdowns (e.g., Scheeren et al.^24^).

Second, the participants tended to be well educated; many already had experience of research participation and were aware of the purpose of the study, which was to explore views on research participation. Our sample may, therefore, have excluded people with less interest in research participation and those with greater support needs. Third, the study did not include people with intellectual disabilities and therefore our sample does not fully represent the autistic community. Fourth, we did not collect data on participants' ethnicity or co-occurring physical or mental health conditions, which meant that we missed on opportunities to add to the diversity of the information captured in the study.

Nevertheless, we purposively sampled our participants to obtain a balanced sample with a wide range of other characteristics and collected data until we felt we achieved information power. Notably, our sample of 31 participants with in-depth 1:1 interview represents a large group when compared with many qualitative studies with the autistic community with typically smaller numbers.

## Conclusion

The COVID-19 lockdown had a varied effect on the lives and routines of autistic people evidenced in their mixed experiences that included seeing opportunities and facing challenges. However, health care providers and researchers need to be mindful of rising mental health issues in the aftermath of the pandemic. The findings suggest that the pandemic may have offered opportunities for innovation in research processes (e.g., wider use of remote methods of consent and data collection), enabling more autistic people to engage with research and making studies more inclusive.

## Supplementary Material

Supplemental data
