# Supplementary material for: Lockdown Experiences and Views on Future Research Participation of Autistic Adults in the UK During the First 6 Months of the COVID-19 Pandemic
Source: Autism Adulthood. 2023 Aug 30;5(3):301–10. doi: 10.1089/aut.2022.0027 (PMC10468553; doi:10.1089/aut.2022.0027)
Supplement: Supplemental data [file Supp_DataS1.docx]

## Lockdown experiences and views on future research participation of autistic adults in the UK during the first six months of the COVID-19 pandemic

## Realpe A, Mills N, Beasant L, Douglas S, Kenny L & Rai D

# SUPPLEMENTAL MATERIAL

## Supplement 1 – Interview Topic Guide

| Part A. Introduction, consent, and background |
| --- |

- Thanks, introduce self, ASK HOW PARTICIPANT WOULD PREFER TO BE ADDRESSED, re-state purpose of the interview
- Aim: to understand what adults with a diagnosis of autism think about participating in a randomised controlled trial (RCT). We will also cover some questions regarding the coronavirus pandemic and how it may impact on research participation.
- Mention recording of interview with permission, data confidentiality/ anonymisation and right to withdrawal at any point without giving a reason
- Ask if any questions on the above
- Record verbal consent to each question on consent form

To start with we would like to briefly ask how, or if, being autistic affects your life, e.g., do you have any routines you like to stick to in everyday life? (Pre-pandemic)

The coronavirus crisis has led to people having to change many aspects of their lives

- How has this affected you and your routines/social interactions (relationships/events)?*[Probe: Thoughts on this - prefer changes or not?]*
- Have your routines changed over the time of lock down? *[Probe: In what way? Why? How feel now compared to earlier in lockdown? Likely to go back to old routines/ways of interacting or stick to new when back to ‘normal’ - why?]*
- How do you think you’ve coped overall with the current situation? *[Probe: Why think this? Always felt like this throughout lockdown or feelings changed? Anything in particular liked or struggled with e.g., social distancing, stay at home?)*
- How does being autistic affect the way you have coped with the current pandemic? *[Probe specific everyday activities: E.g., visiting GP/hospital, supermarket (rules, face coverings, availability food).*
- How much of this is due to autism? Think neurotypical/people without autism might cope differently? *[Probe: anecdotal account of people with chronic anxiety feeling uncharacteristically calm at present because they see it as everyone else joining their way of thinking – can you relate to this or not? Why?]*

There are still so many uncertainties about coronavirus…

- How are you coping with this uncertainty in particular?
- Have you tried any strategies or changes to feel more secure?

| Part B. Views on research studies / current pandemic |
| --- |

I am now going to ask you about your views on research studies, especially in light of the current pandemic:

- Are you the kind of person who would usually volunteer to take part in a research study if you were offered the opportunity? *[Probe: Why? Prior experience? Factors important to make decision (e.g., topic, team expertise, accommodate needs)?]*
- Has this view changed (or might it be likely to change) in any way due to the coronavirus pandemic *[Probe: How? Why?]*
- Do you think researchers need to think about different ways of carrying out research in light of the current pandemic? *[Probe: What sort of things? Why?]*
- What could researchers do to enable autistic people participate in research that may help improve their well-being? *[Probe: thoughts on retaining changes even if not strictly required (e.g., online testing, remote consent)*

| Part C. Understanding RCTs |
| --- |

I’d like to now ask about your understanding of an RCT and your views on it. Before you received information about this study, had you ever heard of a randomised controlled trial (RCT)? It may also be called a randomised clinical trial or study. We sent you a document called “Explaining randomised controlled trials (RCTs)” did you have time to read it?

*General views on participating in RCTs:*

- Does it make sense to you why RCTs are done? Understand the purpose of them?
- Does it make sense why they are done like this? i.e., why treatment is randomised, why an inactive treatment (placebo) may be used, and why the patient and doctor may not know what treatment is given (blinded)?

| Part D. Views on participating in a specific RCT |
| --- |

I’d like to now ask about your views on participating in a specific RCT and then I will ask you some questions about it.

| Researchers would like to find out if a treatment for anxiety (called sertraline) is an effective treatment for anxiety in autistic adults. They know that the treatment works in the non-autistic population, but they do not know if it will treat anxiety in autistic people. They would like to do an RCT to find out. |
| --- |
|  |
| In the RCT, autistic people who have problems with anxiety will be allocated to receive either Sertraline (the real/‘active’ medication) or a ‘Placebo’ (inactive medication). This process is done in a way that everyone has an equal chance of receiving either the active medication or placebo (randomisation). |
|  |
| Neither the person in the study or the doctor will know what medication has been allocated so that perceptions about the medication do not bias the study results (it’s a blinded RCT). |
|  |
| The person in the study will take the sertraline or the placebo for a year without knowing which one it is. |
|  |
| In this time, they will have questionnaires to fill out and they will be closely monitored to check that they are doing ok. They can withdraw from the RCT at any time without giving a reason. |

*Views on participating in a specific RCT:*

- What would you think if you were invited to take part in this RCT?
- What, if anything, might you like about this?
- What, if anything, might concern you about this?
- Do you think you would participate or not? Why?
- What do you think about not knowing what treatment you are taking in a blinded RCT like this?
- Would it make a difference if you didn’t know the treatment you were taking for a short time (perhaps a month) or a longer time (perhaps a year)?
- When the study finishes, and you find out you were in the placebo group – what would you think about that?
- Have you ever taken medication for anxiety or depression (past/currently)? Experience?
- Do you know anyone who in past/currently takes sertraline or medications for anxiety or depression? [from autistic or neuro-typical population?] Their experience?
- Who/what do you trust in terms of getting information about new or existing medications? (GP, Internet etc)

*Views on participating in a specific RCT during the COVID-19 pandemic*

- What would you think about taking part in this RCT during the current coronavirus pandemic?
- What, if anything, might concern you about this? What might you like?
- If social distancing or worries about the pandemic were to continue, would it change your decision to participate or not? Why?
- What could the researchers do in order to facilitate autistic people participate in such a study in the current pandemic?
- Because of social distancing some aspects of the RCT may happen differently e.g., consent, eligibility assessment carried out via video-conference (e.g., skype) or telephone instead of face-to-face. What would you think about this?
- Is there anything else that you would like to raise about taking part in this specific RCT or any research during the current pandemic?

| Part E. Concluding thoughts |
| --- |

- What was it that made you agree to take part in this interview study?
- Is there anything that we have not talked about that you would like to raise?
- Thank them for their time, reiterate confidentiality, discuss reimbursement.

## Supplement 2 – Further illustrative quotes of categories and themes

| **Themes & sub-themes** | **Quotes** |
| --- | --- |
| **Strengths and Resilience** | |
| Enjoying a quieter pace of life | *“I'm quite happy being by myself and not having to go through the ritual of having to understand what people are doing.” P43*  *“All the stressful events were removed and replaced with things I was choosing to do. I settled into it very quickly and enjoyed every minute of it. I was never bored and kept busy with lots of things.” P45* |
| Having a lifestyle amenable to self-isolation | *“I’m very well-suited to COVID life. I spend a lot of [time] at the house by myself anyway. I've got quite insular hobbies, so I like writing, I like painting.” P37*  *“A lot of us tend to be quite solitary people, quite isolated anyway and I think a lot of people, they’re probably experiencing this isolation for the first time.” P23* |
| Being prepared to deal with uncertainty | *“Life carries a risk. And it's your ability to see that and assess it. And I think that's where it helps. Because we [autistic people] have got that. It isn't because we don't empathise. Because we do. We show it in a different way.” P27*  *“The way I deal with the uncertainty is to make as many steps as I can that are within my control (…) I'm happy to follow those rules and to keep informed about the economy. But I know that I can't control those so if I accept those then that's how I deal with the uncertainty.” P36*  *“I tended to look at questions or problems and come out with solutions, which they - people are kind of being a bit left field. I tend to look at things from a different way.” P43*  *“I'm quite matter of fact. So, I don't particularly often have very strong emotions. So, I tend to look at it and look at what the science is saying and what's happening, and take quite a pragmatic view, from that point of view. And I think that's probably due to the autism.” P29* |
| **Challenges adjusting to lockdown** | |
| Changing own lifestyle at pace | *“The first 3 weeks were incredibly difficult. All those micro routines which completely went out of the window, Including the times that I get up. The time that I start and finish work. It completely went out of the window, and it just flawed me. There was no warning (…) So, [lockdown] has had a huge impact because, all those little routines that you have, they create a feeling of safety and wellbeing.” P27*  *“I've got something I want to be doing and can't do it. And I haven't been able to switch to another [leisure activity].” P21* |
| Feeling anxious about work changes | *“My routine was always going to work and coming back home and just chilling out but now they’ve sort of blended into one it’s all been changed quite significantly.” P23*  *“I’m a bit of a loss as to where to go at this stage in terms of finding work, what to do or what would be available.” P49* |
| Missing face-to-face contact | *"So, I've kind of seen a few friends. But I cry when I first see them because I can't hug them. Because I'm a very tactile person. And I really love hugs.” P41*  *“I actually miss the routine of being in work and having a little bit of social contact - I can also close the door if it's too much and I don't have to be around people. But there are people there that I've grown to like.” P42*  *“Time to myself. So, actually, maybe that is an autism thing. I struggle with not having any time to myself.” P20* |
| Feeling anxious about contracting COVID-19 | *“It's the fear of getting the illness for me. We've had three people that we've known that have passed away, which is a bit of a tough one as well. So, it's even closer to home.” P26*  *“My wife finds that she's feeling quite emotional, I can be a bit matter of fact. It's helpful for me. Not necessarily helpful for people I'm living with. I'm not sure I can always give the support (laughs) that she wants.” P29* |
| **Concerns and hopes about end of lockdown** | |
| Increasing uncertainty from lifting of lockdown | *“Even when I try and go out on a walk, I get myself so stressed because people are so blasé, they don't care.” P26*  *“The trouble is that while [lockdown restriction] is a blanket ban, everybody sticks with it. Now there's so many exceptions, it's meaningless. (…) And I think at the moment we're in the worst of both worlds.” P43* |
| Arising mental health issues post-lockdown | *“A month and a half ago I probably would have given you a different answer. I was here on my own with mum and getting no support. And I was finding it very difficult for my mental health.” P33*  *“I think initially I went into kind of crisis mode and for about a month and a half I functioned really well. And I think it hit me in a sort of delayed - So, the past few weeks have been a lot more difficult than even initially.” P48* |
| Hoping for lifting of lockdown at own pace | *“I will be glad when things open up a little bit, I think. And I get back into my own routine a bit, maybe.” P33*  *“I don't mind if the country's getting on enjoying it, but I won't be forced to do anything, so I'll be able to ease into it at my own speed.” P46*  *“I have a lot of things like medical and physical stuff for me and my son (…) And as things go more back to normal, I'm due to have a whole load of stuff now that's had to wait. Suddenly get - actually accelerated a bit and it's that part that I'm worried about - is like now go from this really quite quiet and for me quite easy life to full throttle - back to lots of things to feel anxious and stressed about all the time.” P37* |
| **Views on research participation and engagement** | |
| Relevance of research involvement and participation | *“I think that in general the Coronavirus pandemic has shown that it's important for as many people as possible to participate in studies, if they possibly can, because the bigger your dataset the more accurate your information will be.” P36* |
| Preference for online communication | *“Choice is key here really. Phone, skype, emailing etc. some studies have even been done via Facebook messenger. But each autistic person will want different, so I think it's best not to prescribe a method. And asking autistic people if there's other better ways than have been offered.” P30*  *“Seeing somebody face-to-face is great and I've no problem with that. But for some reason video conferencing is just - urgh. It's really hard work (…) there's a lot of talk about Zoom fatigue.” P27* |
| Support for online research processes | *“But how accessible [the research] is, it is a major one [factor]. If it's online, I'm more likely to do it. I would only do one in person if it were at one of my two local unis within walking distance. And I usually have childcare issues for that, so can only go at certain times.” P40* |
| Predispose to avoid in-person studies | *“I'd expect some kind of physical monitoring.” P20*  *“Any kind of face-to-face thing is going to carry a certain amount of risk.” P29*  *“I'm aware from my knowledge of the history of pandemics that hospitals are not particularly good places to be. One of the pieces of advice to my students was always ‘don't go to hospital if you don't have to’, so I would probably be more wary now of about face-to-face [research activity]” P32*  *“I'm not going to go into a commuter train - not just for my comfort but for my personal safety. So, some things [research activity] change.” P35* |
| Note: P# = Participant unique identification number | |
